# Neoadjuvant Chemotherapy Followed by Gastrectomy for Cytology-Positive Gastric Cancer without Any Other Non-Curative Factors in a Western Setting: An International Eastern European Cohort Study

**DOI:** 10.3390/cancers15245794

**Published:** 2023-12-11

**Authors:** Augustinas Bausys, Toomas Ümarik, Oleksii Dobrzhanskyi, Martynas Luksta, Yourii Kondratskyi, Arvo Reinsoo, Mihhail Vassiljev, Bernardas Bausys, Klaudija Bickaite, Kornelija Rauduvyte, Raminta Luksaite-Lukste, Rimantas Bausys, Kestutis Strupas

**Affiliations:** 1 Clinic of Gastroenterology, Nephrourology, and Surgery, Institute of Clinical Medicine, Faculty of Medicine, Vilnius University, 03101 Vilnius, Lithuania; 2 Centre for Visceral Medicine and Translational Research, Faculty of Medicine, Institute of Clinical Medicine, Vilnius University, 03101 Vilnius, Lithuania; 3 Upper Gastrointestinal Tract Surgery Department, North Estonia Medical Centre, 13419 Tallinn, Estonia; toomas.ymarik@regionaalhaigla.ee (T.Ü.);; 4 Upper Gastrointestinal Tumors Department, National Cancer Institute, 03022 Kyiv, Ukraine; alekseydobrzhanskiy@gmail.com (O.D.);; 5 Pathology Department, North Estonia Medical Centre, 13419 Tallinn, Estonia; 6 Department of Abdominal Surgery and Oncology, National Cancer Institute, 08406 Vilnius, Lithuania

**Keywords:** gastric cancer, positive cytology, neoadjuvant chemotherapy, pathological response

## Abstract

**Simple Summary:**

This multicenter study delved into the outcomes of treating stage IV gastric cancer patients with positive peritoneal cytology but no other non-curative factors using chemotherapy followed by gastrectomy. The findings revealed that preoperative chemotherapy successfully eliminated peritoneal cancer cells in over 50% of patients. The median Overall and Progression-free survival stood at 20 (95% CI: 16–25) and 19 (95% CI: 11–20) months, respectively. Notably, conversion to negative cytology significantly lowered the relative risk of peritoneal progression (RR: 0.11; 95% CI: 0.03–0.47, *p* = 0.002). This study proposes that preoperative chemotherapy followed by gastrectomy shows promise as a viable treatment for stage IV gastric cancer patients with positive peritoneal cytology and no additional non-curative factors. The conversion of cytology status is associated with enhanced long-term outcomes and diminished risk of peritoneal relapse.

**Abstract:**

The optimal approach for treating cytology-positive (Cy1) gastric cancer (GC) patients without additional non-curative factors remains uncertain. While neoadjuvant chemotherapy followed by gastrectomy shows promise, its suitability for Western patients is not well supported by existing data. To address this knowledge gap, a cohort study was conducted across four major GC treatment centers in Lithuania, Estonia, and Ukraine. Forty-three consecutive Cy1 GC patients who underwent neoadjuvant chemotherapy between 2016 and 2020 were enrolled. The study evaluated overall survival (OS), progression-free survival (PFS), cytology status conversion, and major pathological response rates, along with the factors influencing these outcomes. All patients underwent surgery post-neoadjuvant chemotherapy, with 53.5% experiencing cytological status conversion and 23.3% achieving a major pathological response. The median OS and PFS were 20 (95% CI: 16–25) and 19 (95% CI: 11–20) months, respectively. Conversion to negative cytology significantly reduced the relative risk of peritoneal progression (RR: 0.11; 95% CI: 0.03–0.47, *p* = 0.002). The study suggests that neoadjuvant chemotherapy followed by gastrectomy holds promise as a treatment option for Cy1 GC without additional non-curative factors, associating cytology status conversion with improved long-term outcomes and reduced peritoneal relapse risk.

## 1. Introduction

Gastric cancer (GC) ranks among the most prevalent malignancies globally, with over 1 million new cases and 750 thousand annual deaths [1]. Surgery remains the primary and only curative treatment option [2,3]. Unfortunately, up to 40% of patients present with distant metastases at the time of diagnosis, rendering them ineligible for radical surgery [4,5]. The peritoneum is the most frequent site of distant metastases [6,7]. Staging laparoscopy coupled with peritoneal lavage cytology stands as the diagnostic standard for detecting early peritoneal dissemination when only free cancer cells are present and peritoneal carcinomatosis (P1) has not yet formed [8,9,10,11]. Positive peritoneal cytology (Cy1), irrespective of other non-curative factors, emerges as a robust negative prognostic indicator for recurrence and survival [12]. Consequently, it is categorized as distant metastases (M1) and Cy1 patients are classified as stage IV, regardless of other non-curative factors [6].

Presently, there exists no international consensus on the optimal treatment for Cy1 GC patients. Western guidelines advocate for palliative care with potential re-staging post treatment [6,13]. In contrast, Eastern guidelines identify Cy1 patients as a distinct subset within the stage IV cohort, proposing the consideration of neoadjuvant chemotherapy followed by D2 gastrectomy if no other non-curative factors are present [14]. These disparities in recommendations and the absence of a widely accepted treatment for Cy1 patients stem from a knowledge gap. Hence, this study aims to explore the outcomes of a neoadjuvant approach for Cy1 GC within a Western cohort.

## 2. Material and Methods

### 2.1. Ethics

Local ethics committees or institutional review boards approved the study in each center before this study was conducted. All study-related procedures were performed following the Declaration of Helsinki of 1975, as revised in 1983.

### 2.2. Patients and Diagnostic Pathway

**Figure 1:**
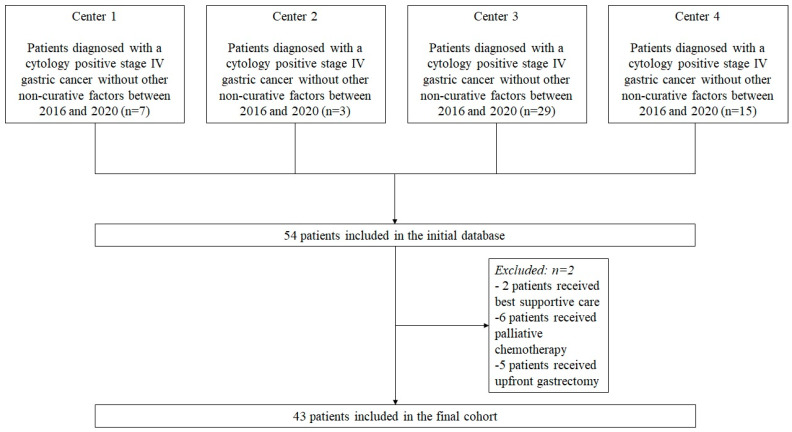
Flowchart of the study patients.

This cohort study screened all consecutive patients who were diagnosed with Cy1 stage IV GC without any other distant metastases between January 2016 and December 2020. The study was conducted at four major upper gastrointestinal cancer treatment centers in Lithuania, Estonia, and Ukraine: (1) National Cancer Institute, Vilnius, Lithuania; (2) Vilnius University hospital Santara Clinics, Vilnius, Lithuania; (3) National Cancer Institute, Kyiv, Ukraine; (4) North Estonia Medical Centre, Tallinn, Estonia. The standard diagnostic pathway for gastric cancer patients was consistent with current European Society for Medical Oncology (ESMO) guidelines [13] and included endoscopy with biopsy followed by chest and abdominal computed tomography (CT). If ≥cT2 or N+ disease and no distant metastases were detected at preoperative imaging, patients underwent diagnostic laparoscopy with peritoneal lavage for cytology. In the case of any suspicious peritoneal lesions, biopsies were taken to confirm or rule out peritoneal carcinomatosis (P1). After Cy1 GC without other non-curative factors was confirmed, all patients were discussed in multidisciplinary team meetings. Patients who were allocated to receive treatment with neoadjuvant chemotherapy followed by gastrectomy were included in the study; those who were allocated to receive best supportive care, upfront gastrectomy, or palliative chemotherapy were excluded (Figure 1).

### 2.3. Treatment and Follow-Up of Study Patients

The standard neoadjuvant treatment consisted of 3–6 cycles of chemotherapy; the exact number of cycles and regimens was selected by a medical oncologist. After neoadjuvant chemotherapy was completed, patients were scheduled for chest and abdominal CT and, if no distant metastases were detected, patients were scheduled for gastrectomy. An open or laparoscopic approach was selected by a surgeon. Subtotal gastrectomy was performed if a sufficient proximal resection margin could be ensured; otherwise, total gastrectomy was performed. In the case of an unresectable primary tumor, palliative procedures were performed if necessary. The extent of lymphadenectomy depended on the individual surgeon’s decision, but the standard lymphadenectomy was a D2 lymph node dissection performed as described in the 6th version of Japanese gastric cancer treatment guidelines [14]. All patients were considered for adjuvant chemotherapy after recovery from surgery. The standard follow-up protocol consisted of CT every 3 months for the first 2 years and, later, biannually. Also, esophagogastroduodenoscopy was performed 1 year after surgery.

### 2.4. Study Outcomes

The primary outcome of the study was overall survival (OS). Secondary end-points were progression-free (PFS) survival; conversion to negative cytology after neoadjuvant chemotherapy rates; major pathological response (mPR) after neoadjuvant chemotherapy rates; and postoperative complication rates. All postoperative complications were graded by Clavien–Dindo classification, and severe complications were defined as grade ≥3. OS was defined as the time between diagnosis of Cy1 stage IV GC and death. PFS was defined as the time between diagnosis of Cy1 GC and progression of the disease or death.

### 2.5. Statistical Analysis

All statistical analyses were conducted using the statistical program SPSS 25.0 (SPSS, Chicago, IL, USA). Continuous variables were presented as medians within the first (Q1) and third (Q3) quartiles. These variables were compared across groups using the Mann–Whitney U test or the Kruskal–Wallis test. Categorical variables were shown as proportions and were compared using the Chi-square test or Fisher’s exact test. OS and PFS rates were analyzed using the Kaplan–Meier method and were compared between the study groups using the log-rank test. To identify the factors impacting long-term outcomes in the neoadjuvant approach group, multivariable Cox proportional hazards regression analysis was used. Hazard ratios (HRs) were presented with 95% confidence intervals (CI). In all statistical analyses, two-tailed tests were used and a *p*-value of <0.05 was considered to be significant. The listwise deletion method was used for missing data.

## 3. Results

### 3.1. Baseline Characteristics and Neoadjuvant Chemotherapy

In total, 43 participants, with a median age of 57 (45; 65) years, were enrolled in the study. Each participant underwent a median of four (three; six) cycles of neoadjuvant chemotherapy. The most common chemotherapy regimen was fluorouracil, folinic acid, oxaliplatin, and docetaxel (FLOT), administered to 26 (60.5%) patients (Table 1).

### 3.2. Outcomes of Surgical Treatment, Cytological Status Conversion, and Major Pathological Response Rates

After completing neoadjuvant treatment, all patients underwent surgery. Palliative procedures were conducted in 3 (7.0%) patients, while another 40 (93.0%) patients underwent total or subtotal gastrectomy accompanied by D2 lymphonodectomy in 35 patients (87.5%) (Table 2). Postoperative complications occurred in 19 (45.2%) patients, including severe complications (Clavien–Dindo ≥ 3) in 9 (21.4%) patients.

**Figure 2:**
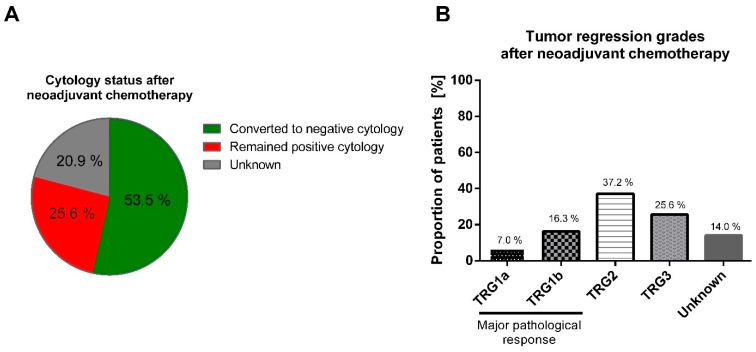
Neoadjuvant chemotherapy impact on the cytological status and pathological response in the primary tumor. After neoadjuvant chemotherapy, 53.5% of patients converted from positive to negative cytology (**A**); major pathological response by TRG1a/b was achieved by 23.3% of patients (**B**). TRG: tumor regression grade by Becker classification.

Post-surgery, cytological and histological examinations indicated that 23 patients (53.5%) experienced a conversion to negative cytology, and 10 patients (23.3%) achieved a major pathological response (mPR), classified as TRG1a/1b by Becker, following neoadjuvant treatment (Figure 2). Notably, there was no observed correlation between conversion to negative cytology and the achievement of a major pathological response (R = −0.302; *p* = 0.119). Further, there were no differences between patients who converted to negative cytology and those who maintained a positive cytology in terms of sex, age, ECOG score, tumor localization, cT, cN, presence of signet ring cells, lymphovascular invasion, and HER2 status, *p* > 0.05.

The type of neoadjuvant chemotherapy, along with patient and tumor characteristics, did not show associations with the rates of conversion to negative cytology or mPR (Table 3). However, clinically negative lymph nodes were associated with higher odds (OR: 29; 95% CI: 4–210) of achieving mPR. After surgical treatment, 26 (61.9%) patients underwent adjuvant chemotherapy.

### 3.3. Long-Term Outcomes

**Figure 3:**
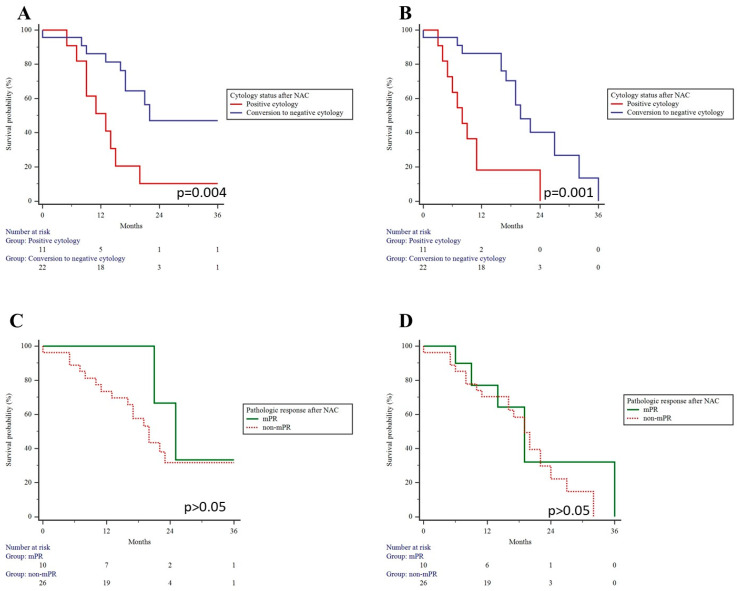
Overall and progression-free survival in patients who converted to negative cytology and achieved major pathological response after neoadjuvant chemotherapy. Conversion to negative cytology resulted in better overall (**A**) and progression-free survival (**B**). Major pathological response had no impact on overall (**C**) and progression-free survival (**D**) rates.

The median follow-up time was 16 (9; 21) months. Univariate Kaplan–Meier analysis revealed a median OS and PFS of 20 (95% CI: 16–25) and 19 (95% CI: 11–20) months, respectively. Notably, the conversion to negative cytology after neoadjuvant chemotherapy was linked to improved OS and PFS, whereas an mPR did not significantly impact long-term outcomes (Figure 3).

Throughout the follow-up period, a total of 12 patients (27.3%) were diagnosed with peritoneal metastasis, representing the most common site of progression. Peritoneal recurrence was almost exclusively observed in patients who retained positive cytology after neoadjuvant chemotherapy (72.7% vs. 8.7%, *p* = 0.001). Conversion to negative cytology significantly reduced the relative risk for peritoneal progression (RR: 0.11; 95% CI: 0.03–0.47, *p* = 0.002). Additionally, multivariable Cox regression analysis demonstrated that conversion to negative cytology after neoadjuvant chemotherapy correlated with a decreased risk of death (HR: 0.05; 95% CI: 0.01–0.58; *p* = 0.017) and recurrence (HR: 0.10; 95% CI: 0.01–0.68; *p* = 0.019) after adjusting for age, mPR, type of chemotherapy, pathologic tumor, and nodal status (Table 4).

## 4. Discussion

This study elucidates the short- and long-term outcomes in Cy1 GC patients without additional non-curative factors following treatment with neoadjuvant chemotherapy. After neoadjuvant chemotherapy, 53.5% of Cy1 patients experienced a conversion to negative cytology, and 23.3% achieved a major pathological response. Importantly, the conversion in cytologic status was linked to a significant reduction in the risk of death and recurrence, and particularly a lower risk for peritoneal relapse.

Treatment for Cy1 GC patients lacks standardization due to the absence of high-quality evidence. Free cancer cells detectable on cytology from peritoneal lavage signify peritoneal dissemination and metastatic disease. Consequently, akin to other GC metastases, palliative chemotherapy emerges as a standard treatment option. Unfortunately, systemic chemotherapy exhibits limited efficacy for GC peritoneal lesions [15], yielding a median survival of only 7 months [16]. Given the unsatisfactory long-term outcomes and distinct differences between Cy1 patients and GC patients with macroscopic carcinomatosis, more aggressive treatment strategies, including surgery, may be considered. Among treatments involving gastrectomy, two different options exist: upfront gastrectomy followed by adjuvant chemotherapy and gastrectomy after neoadjuvant chemotherapy. The CCOG0301 phase II single-arm study demonstrated that upfront gastrectomy followed by adjuvant S-1 monotherapy achieved 5-year OS and relapse-free survival rates of 26% and 21%, respectively. However, the peritoneal recurrence rate after such treatment is notably high at 62% [17]. Similar outcomes for upfront gastrectomy were confirmed in a retrospective study by Kano et al., revealing a 5-year OS of 17.8% and a peritoneal recurrence rate of 52.9% [18]. Further, a recent retrospective study by Bailong et al. demonstrated comparable survival outcomes for patients who underwent upfront gastrectomy and those who had gastrectomy after neoadjuvant treatment [19]. Nevertheless, the long-term outcomes achieved by preceding gastrectomy may be significantly compromised if patients do not receive adjuvant chemotherapy. Adjuvant chemotherapy after gastrectomy for Cy1 CG patients enhances OS to 22–25 months compared to 11–12 months in patients undergoing only surgical treatment [18,20]. However, the inability to tolerate cytotoxic treatment after major surgery, such as gastrectomy, is a serious issue, as 36% of patients are unable to receive adjuvant treatment due to the deterioration of their general condition after gastrectomy. This percentage can further rise to about 63% in the case of severe postoperative complications [21]. In contrast, chemotherapy applied in a neoadjuvant setting is better tolerated, with a compliance rate of more than 90% [22]. This difference may favor the neoadjuvant approach. The present study demonstrates that treatment with neoadjuvant chemotherapy followed by gastrectomy achieves acceptable long-term outcomes, with a median OS of 20 months (95% CI: 16–25). Neoadjuvant chemotherapy downsized the disease by converting to negative cytology in 53.5% of patients, and this conversion was associated with a significantly decreased risk of death (HR: 0.05; 95% CI: 0.01–0.58; *p* = 0.017) and recurrence (HR: 0.10; 95% CI: 0.01–0.68; *p* = 0.019). Our present findings align with results from previous small-scale studies, demonstrating improved long-term outcomes in 48.9–72.2% of Cy1 patients who achieve cytology status conversion [23,24,25,26]. Poor long-term outcomes in those who remain positive on cytology underscore the necessity for re-evaluation with diagnostic laparoscopy after neoadjuvant chemotherapy, because it may help to avoid almost half of the surgeries resulting in R1 resection. Furthermore, our study reveals that the vast majority of patients (72.7%) who remain positive on cytology after neoadjuvant chemotherapy will eventually develop peritoneal carcinomatosis. Considering that current systemic chemotherapy does not benefit these patients, it is crucial to explore and embrace new biomarkers. These biomarkers would play a key role in predicting the response to systemic neoadjuvant chemotherapy and allowing for personalized treatment for every patient [27]. This becomes particularly important as alternative treatment modalities, like intraperitoneal cytotoxic therapy, emerge as potential options for patients. A pilot study by Imano et al. showed that 80 mg/m^2^ paclitaxel applied intraperitoneally at the end of radical D2 gastrectomy can clear peritoneal cytology. Moreover, this study showed a promising 3-year survival rate of 56% and a peritoneal recurrence rate of 30% [28]. However, conflicting data exist on the effectiveness of intraperitoneal chemotherapy. A randomized controlled study from Japan showed a poor 5-year OS of 4.6% and 0% in patients who received gastrectomy and intraperitoneal chemotherapy with 100 mg cisplatin or gastrectomy alone. Thus, this approach remains controversial. Interestingly, the same study demonstrated promising outcomes with a 5-year OS rate of 43.8% in patients who received extensive peritoneal lavage with 10 L of a saline solution together with gastrectomy and intraperitoneal chemotherapy. Furthermore, intraperitoneal lavage reduced the peritoneal progression rate to 40.0% compared to 79.3% in the IPC group and 89.7% in the group receiving gastrectomy alone [29]. However, these techniques are rare outside of East Asia and would be considered experimental treatment in West.

Another available option for peritoneal disease, including GC, is hyperthermic intraperitoneal chemotherapy (HIPEC). A recent meta-analysis of randomized and high-quality non-randomized trials showed that HIPEC had no impact on long-term outcomes in GC patients with peritoneal carcinomatosis but may have a role in a prophylactic setting. HIPEC reduces the risk of peritoneal metastases (RR = 0.63; 95% CI: 0.45–0.88; *p* < 0.01) in high-risk patients, including Cy1 GC patients [30]. HIPEC can also find application in a neoadjuvant setting. A phase II study conducted by Badgwell et al. revealed that administering five cycles of neoadjuvant laparoscopic HIPEC after initial systemic chemotherapy resulted in cytology status conversion in 66.6% of patients [31]. However, this conversion rate does not significantly surpass the 53.5% achieved in our study with neoadjuvant chemotherapy alone. The broader acceptance of HIPEC for Cy1 GC patients is hindered by the scarcity of data from high-quality randomized controlled trials. The ongoing GASTRICHIP study, which explores the use of HIPEC in patients at high risk of peritoneal recurrence, including Cy1 patients after neoadjuvant chemotherapy, is anticipated to contribute more data to the field [32]. Another innovative technique for delivering chemotherapy intraperitoneally for GC peritoneal metastases is pressurized intraperitoneal chemotherapy (PIPAC) [33]. However, there are a lack of data regarding its efficacy, specifically in Cy1 patients.

The current study has some limitations that have to be considered. Firstly, being a retrospective study, it inherently carries typical disadvantages, including the potential for selection bias. Participants were chosen based on their eligibility for neoadjuvant chemotherapy, possibly excluding individuals with specific characteristics or conditions. Secondly, the relatively small sample size could impact the statistical power of the study, making it challenging to discern subtle differences in outcomes. Thirdly, the study’s single-arm design, focusing on the neoadjuvant approach, lacks a robust comparison with alternative treatment modalities like upfront gastrectomy or palliative care. This limitation restricts the assessment of the relative effectiveness of different strategies. Notably, the low number of patients treated with alternative methods in our initial database (n = 6 palliative chemotherapy; n = 5 upfront gastrectomy) precluded their inclusion for meaningful comparison. Fourthly, the median follow-up time of 16 months might not suffice to capture long-term outcomes and evaluate the enduring efficacy of the neoadjuvant treatment strategy. Longer follow-up durations would offer a more comprehensive understanding of survival and recurrence patterns. Fifthly, our present study exclusively involved patients of the Caucasian race from Lithuania, Estonia, and Ukraine. Consequently, the generalization of our findings to other Western cohorts may be somewhat restricted. Despite these limitations, it is crucial to interpret the findings cautiously and underscore the necessity for further research to address these constraints. Notably, this study represents the largest cohort of Western patients, showcasing the efficacy of the neoadjuvant approach in Cy1 GC patients given the current knowledge landscape.

## 5. Conclusions

In conclusion, this study provides novel evidence that neoadjuvant chemotherapy followed by gastrectomy is a promising treatment option for cytology-positive gastric cancer patients without other non-curative factors in a Western setting. Clearance of cytology is associated with improved outcomes and a lower risk for peritoneal relapse; thus, cytological status re-evaluation should be standard before considering radical surgery.

## Figures and Tables

**Table 1:** Baseline characteristics of study patients.

Characteristics		Patients (n = 43)
Age; median (Q1; Q3), years		57 (45; 65)
Sex; n (%)	Male	22 (51.2%)
	Female	21 (48.8%)
CCI; median (Q1; Q3)		5 (3; 7)
ECOG score; n (%)	0–1	42 (97.7%)
	≥2	1 (2.3%)
Tumor localization; n (%)	Cardia	11 (25.6%)
	Body	16 (37.2%)
	Antrum	9 (20.9%)
	Linitis Plastica	7 (16.3%)
cT; n (%)	T1-2	6 (14.0%)
	T3-4	37 (86.0%)
cN; n (%)	N0	9 (20.9%)
	N+	34 (79.1%)
Signet ring cell; n (%)	Yes	18 (42.9%)
	No	24 (57.2%)
Lymphovascular invasion; n (%)	Yes	24 (61.5%)
	No	15 (38.5%)
Lauren type; n (%)	Diffuse	18 (42.9%)
	Mix	2 (2.3%)
	Intestinal	23 (54.8%)
HER2 status; n (%)	Negative	35 (92.1%)
	Positive	3 (7.9%)
Type of chemotherapy; n (%)	FLOT	26 (60.5%)
	FOLFOX/XELOX/other platinum- and fluorouracil-based duplet	13 (30.2%)
	ECX/EOX	4 (9.3%)

Q1: quartile 1; Q3: quartile 3; CCI: Charlson Comorbidity Index; ECOG: Eastern Cooperative Oncology Group; cT: clinical tumor stage according to TNM classification; cN: clinical nodal stage according to TNM classification; FLOT: fluorouracil, folinic acid, oxaliplatin, and docetaxel; FOLFOX: fluorouracil, folinic acid, and oxaliplatin; ECX: epirubicin, cisplatin, capecitabine; EOX: epirubicin, oxaliplatin, capecitabine; XELOX: oxaliplatin and capecitabine.

**Table 2:** Surgical treatment outcomes in study patients.

Characteristics		Patients (n = 43)
Type of surgery; n (%)	Total gastrectomy	35 (81.4%)
	Subtotal gastrectomy	5 (11.6%)
	Palliative procedure	3 (7.0%)
Lymphadenectomy; n (%)	D1	5 (12.5%)
	D2	35 (87.5%)
Surgical approach	Open	39 (92.9%)
	Laparoscopic	3 (7.1%)
Multiorganic resection; n (%)	No	25 (59.5%)
	Yes	17 (40.5%)
Length of surgery, minutes (median; (Q1; Q3))		227 (163; 298)
R; n (%)	R0–1	42 (97.7%)
	R2	1 (2.3%)
Retrieved LN number (median; (Q1; Q3))		26 (20; 33)
Postoperative complications (any); n (%)		19 (45.2%)
Type of complications, n (%)	Anastomotic leakage	2 (4.6%)
	Pancreatic fistula/pancreatitis	2 (4.6%)
	Pulmonary complications	7 (16.2%)
	Wound infection or intraabdominal abscess	2 (4.6%)
	Other	6 (13.9%)
Severe postoperative complications (Clavien–Dindo ≥ 3); n (%)		9 (21.4%)
Intrahospital or 30 days postoperative mortality rate; n (%)		3 (7.1%)

Q1: quartile 1; Q3: quartile 3; R: residual tumor.

**Table 3:** Factors associated with conversion to negative cytology and major histologic tumor regression after neoadjuvant chemotherapy.

Variable		Proportion of Patients Converting to Negative Cytology, n (%)	*p* Value	Proportion of Patients with Major Histologic Tumor Regression, n (%)	*p* Value
Sex	Male	5 (26.3%)	0.397	7 (36.8%)	0.269
	Female	6 (40.0%)		3 (16.7%)	
Age	≤60	5 (23.8%)	0.176	8 (40.0%)	0.073
	>60	6 (46.2%)		2 (11.8%)	
cT	cT1-2	3 (60%)	0.152	3 (75.0%)	0.052
	cT3-4	8 (27.6%)		7 (21.2%)	
cN	cN0	0 (0%)	0.150	7 (77.8%)	0.001
	cN+	11 (32.4%)		3 (10.7%)	
Tumor localization	Cardia	3 (33.3%)	0.195	3 (33.3%)	0.151
	Body	5 (45.5%)		5 (35.7%)	
	Antrum	3 (42.9%)		2 (25.0%)	
	Linitis plastica	0 (0%)		0 (0%)	
Type of chemotherapy	FLOT	7 (31.8%)	0.999	7 (30.4%)	0.710
	Other *	4 (33.3%)		3 (21.4%)	
Tumor differentiation grade	G1-2	4 (20.0%)	0.217	7 (31.8%)	0.709
	G3	5 (45.5%)		3 (23.1%)	
Type by Lauren classification	Diffuse	4 (33.3%)	0.999	3 (20.0%)	0.480
	Intestinal/Mix	6 (28.6%)		7 (31.8%)	
Signet ring cell carcinoma	Yes	6 (46.2%)	0.139	4 (25.0%)	0.999
	No	4 (20%)		6 (28.6%)	
Lymphovascular invasion	Yes	4 (26.7%)	0.999	6 (26.1%)	0.999
	No	3 (20%)		4 (28.6%)	
HER2 status	Positive	1 (33.3%)	0.999	2 (66.7%)	0.201
	Negative	6 (22.2%)		8 (25.8%)	

cT: clinical tumor stage according to TNM classification; cN: clinical nodal stage according to TNM classification; FLOT: fluorouracil, folinic acid, oxaliplatin, and docetaxel; *: other types of chemotherapy; FOLFOX: fluorouracil, folinic acid, and oxaliplatin; ECX: epirubicin, cisplatin, capecitabine; EOX: epirubicin, oxaliplatin, capecitabine; XELOX: oxaliplatin and capecitabine.

**Table 4:** Multivariable Cox regression analysis for overall and disease-free survival.

Variable	Category	Overall Survival		Disease-Free Survival	
		HR (95% CI)	*p* Value	HR (95% CI)	*p* Value
Age		0.89 (0.82–0.98)	0.018	0.97 (0.92–1.03)	0.371
mPR	Non-mPR	1 (Reference)		1 (Reference)	
	mPR	0.54 (0.04–6.12)	0.625	1.03 (0.11–9.56)	0.974
Cytology status after neoadjuvant chemotherapy	Positive cytology	1 (Reference)		1 (Reference)	
	Conversion to negative cytology	0.05 (0.01–0.58)	0.017	0.10 (0.01–0.68)	0.019
Type of chemotherapy	Non-FLOT	1 (Reference)		1 (Reference)	
	FLOT	0.11 (0.01–0.96)	0.046	0.48 (0.09–2.42)	0.482
ypT	ypT3-4	1 (Reference)		1 (Reference)	
	ypT1-2	0.01 (0.01–0.29)	0.007	0.04 (0.01–0.58)	0.018
ypN	ypN+	1 (Reference)		1 (Reference)	
	ypN0	0.69 (0.11–4.34)	0.694	0.41 (0.07–2.43)	0.331

HR: hazard ratio; 95% CI: 95% confidence interval; mPR: major pathological response; FLOT: fluorouracil, folinic acid, oxaliplatin, and docetaxel; ypT: pathologic tumor stage after neoadjuvant chemotherapy according to TNM classification; ypN: pathologic nodal stage after neoadjuvant chemotherapy according to TNM classification.

## Data Availability

The data presented in this study are available on reasonable request from the corresponding author.
